# Subtle Variations in Dietary-Fiber Fine Structure Differentially Influence the Composition and Metabolic Function of Gut Microbiota

**DOI:** 10.1128/mSphere.00180-20

**Published:** 2020-05-06

**Authors:** Yunus E. Tuncil, Riya D. Thakkar, Seda Arioglu-Tuncil, Bruce R. Hamaker, Stephen R. Lindemann

**Affiliations:** a Food Engineering Department, Ordu University, Ordu, Turkey; b Whistler Center for Carbohydrate Research, Department of Food Science, Purdue University, West Lafayette, Indiana, USA; c Department of Nutrition Science, Purdue University, West Lafayette, Indiana, USA; d Gastronomy and Culinary Arts Department, Bitlis Eren University, Ahlat, Bitlis, Turkey; University of Michigan-Ann Arbor

**Keywords:** wheat, arabinoxylan, carbohydrate, linkage, monosaccharide, colonic microbiome short-chain fatty acids, 16S rRNA, *Bacteroides*, *Prevotella*, carbohydrate structure, dietary fiber

## Abstract

The chemical structures of soluble fiber carbohydrates vary from source to source due to numerous possible linkage configurations among monomers. However, it has not been elucidated whether subtle structural variations might impact soluble fiber fermentation by colonic microbiota. In this study, we tested the hypothesis that subtle structural variations in a soluble polysaccharide govern the community structure and metabolic output of fermenting microbiota. We performed *in vitro* fecal fermentation studies using arabinoxylans (AXs) from different classes of wheat (hard red spring [AX_HRS_], hard red winter [AX_HRW_], and spring red winter [AX_SRW_]) with identical initial microbiota. Carbohydrate analyses revealed that AX_SRW_ was characterized by a significantly shorter backbone and increased branching compared with those of the hard varieties. Amplicon sequencing demonstrated that fermentation of AX_SRW_ resulted in a distinct community structure of significantly higher richness and evenness than those of hard-AX-fermenting cultures. AX_SRW_ favored OTUs within *Bacteroides*, whereas AX_HRW_ and AX_HRS_ favored *Prevotella*. Accordingly, metabolic output varied between hard and soft varieties; higher propionate production was observed with AX_SRW_ and higher butyrate and acetate with AX_HRW_ and AX_HRS_. This study showed that subtle changes in the structure of a dietary fiber may strongly influence the composition and function of colonic microbiota, further suggesting that physiological functions of dietary fibers are highly structure dependent. Thus, studies focusing on interactions among dietary fiber, gut microbiota, and health outcomes should better characterize the structures of the carbohydrates employed.

**IMPORTANCE** Diet, especially with respect to consumption of dietary fibers, is well recognized as one of the most important factors shaping the colonic microbiota composition. Accordingly, many studies have been conducted to explore dietary fiber types that could predictably manipulate the colonic microbiota for improved health. However, the majority of these studies underappreciate the vastness of fiber structures in terms of their microbial utilization and omit detailed carbohydrate structural analysis. In some cases, this causes conflicting results to arise between studies using (theoretically) the same fibers. In this investigation, by performing *in vitro* fecal fermentation studies using bran arabinoxylans obtained from different classes of wheat, we showed that even subtle changes in the structure of a dietary fiber result in divergent microbial communities and metabolic outputs. This underscores the need for much higher structural resolution in studies investigating interactions of dietary fibers with gut microbiota, both *in vitro* and *in vivo*.

## Abstract (precis)

Diet, especially with respect to consumption of dietary fibers, is well recognized as one of the most important factors shaping the colonic microbiota composition. Accordingly, many studies have been conducted to explore dietary fiber types that could predictably manipulate the colonic microbiota for improved health. However, the majority of these studies underappreciate the vastness of fiber structures in terms of their microbial utilization and omit detailed carbohydrate structural analysis. In some cases, this causes conflicting results to arise between studies using (theoretically) the same fibers. In this investigation, by performing *in vitro* fecal fermentation studies using bran arabinoxylans obtained from different classes of wheat, we showed that even subtle changes in the structure of a dietary fiber result in divergent microbial communities and metabolic outputs. This underscores the need for much higher structural resolution in studies investigating interactions of dietary fibers with gut microbiota, both *in vitro* and *in vivo*.

## OBSERVATION

Although plant carbohydrates are composed of a relatively limited set of different monosaccharides, astronomical structural diversity arises from the multiplicity of anomeric configurations, linkage types, backbone lengths, branching units, and reducing terminal attachments that occur in these often-branched polymers (1–5). Further, as biosynthetic enzymes generating plant cell wall polysaccharides are sometimes imprecise, these reactions can be viewed to generate a family of related structural features that can vary significantly among plant species or even different tissues within a plant (1). Conversely, microbial hydrolases are often quite structure specific in their degradative activities (6), requiring a large complement of distinct enzymes to degrade complex polysaccharides and limiting microbial access to such carbohydrates (7). However, this structural diversity is often overlooked when examining the effects of fiber carbohydrates on the community structure and metabolic function of human gut microbiota, raising the question of whether distinct structural variants might interact differently. Recently, the fine structure of insoluble starch molecules was found to influence gut microbiome structural and functional responses in a dose-dependent way in a human feeding study (8). This suggests tight interrelationships between physical and chemical structuring and the organisms most competitive in exploiting these differences (8). In this study, we tested the hypothesis that subtle variation in a soluble fiber carbohydrate’s chemical structure would govern the outcome of microbial competition, using arabinoxylan (AX) hemicelluloses extracted from brans of three different commonly consumed classes of wheat (Triticum aestivum)—hard red spring (AX_HRS_), hard red winter (AX_HRW_), and soft red winter (AX_SRW_)—as model fiber carbohydrates.

**FIG 1:**
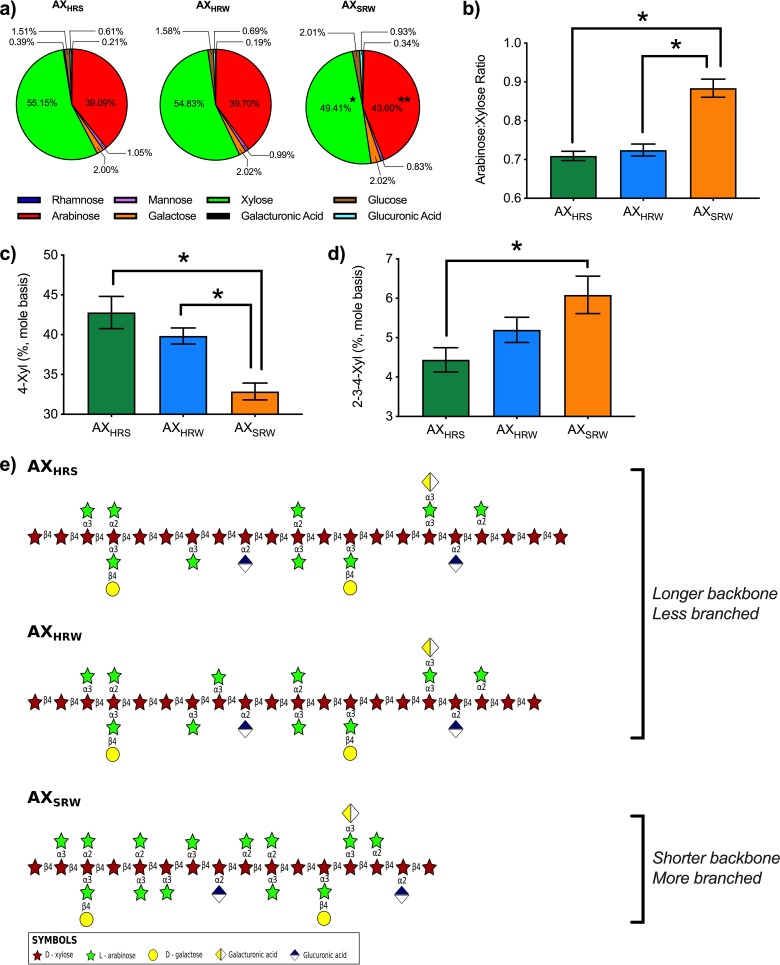
Compositional and structural features of AXs used. (a) Monosaccharide compositions (mole basis) (*, significantly smaller amount of xylose; **, significantly larger amount of arabinose; two-tailed Student’s *t* test, *P < *0.05). (b) Arabinose-to-xylose ratio (as an indicator of branching density). (c) Relative abundances of 4-Xyl linkage (typical linkage presented in the backbone of AX). (d) Relative abundances of 2-3-4-Xyl linkage (typical linkage presented in the backbone of AX that bear side chains) (other linkages detected are given in Table S1). (e) Schematic of proposed generalized structures of the AX samples drawn based on monosaccharide compositions and linkage profiles. Arabinoxylan extracted from hard red spring wheat (AX_HRS_) and arabinoxylan extracted from hard red winter wheat (AX_HRW_) have longer backbones and fewer branching points than arabinoxylan extracted from soft red winter wheat (AX_SRW_). Statistical analyses were done using two-tailed Student’s *t* test (*P < *0.05). Error bars represent the standard errors of three separate replicates.

We observed no significant differences in the starch content, molecular weight, or dispersity values of the extracted AXs (*P > *0.05) (see Fig. S1 in the supplemental material). AX_HRS_ and AX_HRW_ were almost identical in sugar composition, displaying no significant differences in any of their acidic and neutral monosaccharides (Fig. 1a). However, AX_SRW_ exhibited a significantly higher abundance of arabinose and a lower abundance of xylose (*P < *0.05) (Fig. 1a). Xylose and arabinose are the main building blocks of AX; the former composes the linear backbone and the latter the major branches of the AX. Thus, the arabinose-to-xylose ratio provides an estimate of an AX’s branching density. The arabinose-to-xylose ratios of AX_HRS_ and AX_HRW_ were not distinguishable, whereas that of AX_SRW_ was significantly higher (Fig. 1b), indicating an increasingly branched structure compared to those of AX_HRS_ and AX_HRW_. Examination of the glycosyl linkages (Table S1) further supports significant differences in branch structures. AX_HRS_ and AX_HRW_ exhibited a significantly higher proportion of 4-xylose linkages (Fig. 1c), which join the xylose units in the linear backbone of AXs. Conversely, a greater abundance of the branch-associated 2-3-4-linked xylose was observed in AX_SRW_ (*P < *0.05) (Fig. 1d). Collectively, these data indicate that AX_SRW_ has a shorter backbone and increased branch density compared to those of AX_HRS_ and AX_HRW_ (Fig. 1e). It should be noted that we did not quantify the protein contents of the AXs, as extensive enzyme (proteases pepsin and pancreatin) treatments applied during the extraction and mimic upper gastrointestinal tract digestion are considered to be sufficient to remove proteins (Text S1). Similarly, ferulic acid contents of the AXs were not quantified, as alkali-extracted wheat bran AXs have been shown to possess no detectable ferulic acid (9). However, as the polysaccharides were extracted from intact wheat brans, it cannot be ruled out that retained traces of these or other components may have influenced the observed fermentation dynamics.

10.1128/mSphere.00180-20.1 TEXT S1 Materials and methods. Download Text S1, DOCX file, 0.04 MB. Copyright © 2020 Tuncil et al. 2020 Tuncil et al. This content is distributed under the terms of the Creative Commons Attribution 4.0 International license.

10.1128/mSphere.00180-20.2 FIG S1 Starch contents, molecular sizes, and dispersity values of the AXs used. Statistical analyses were done using two-tailed Student’s *t* test. Error bars represent the standard errors of three separate replicates. ns, not significant. Download FIG S1, TIF file, 0.6 MB. Copyright © 2020 Tuncil et al. 2020 Tuncil et al. This content is distributed under the terms of the Creative Commons Attribution 4.0 International license.

10.1128/mSphere.00180-20.10 TABLE S1 Linkage compositions of AXs (percent, mole basis). Download Table S1, DOCX file, 0.08 MB. Copyright © 2020 Tuncil et al. 2020 Tuncil et al. This content is distributed under the terms of the Creative Commons Attribution 4.0 International license.

**FIG 2:**
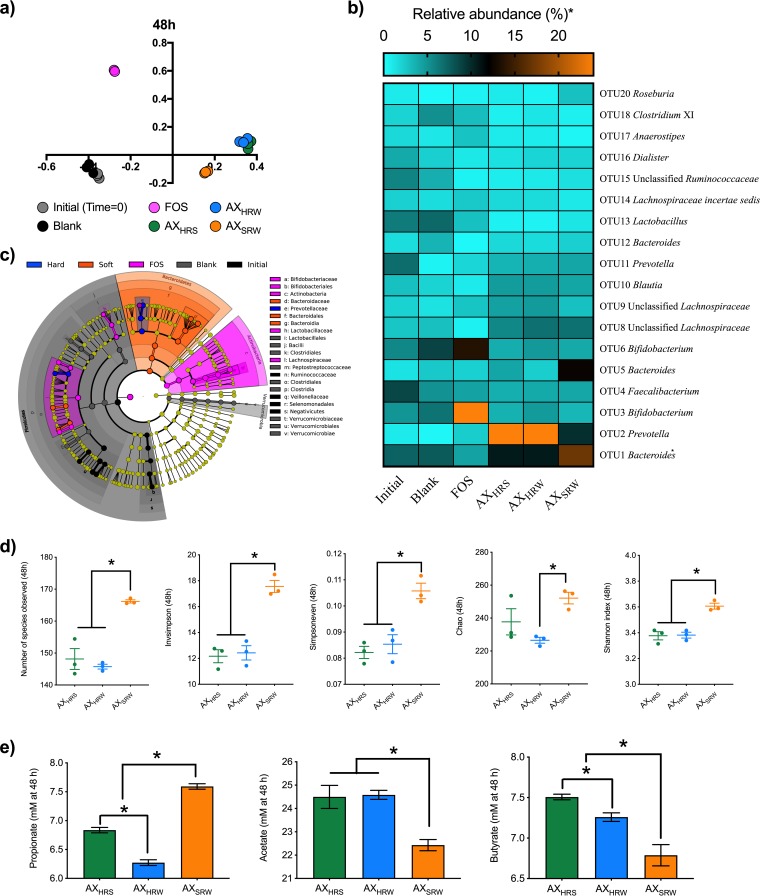
Microbial community analyses after *in vitro* fermentation for 48 h, as determined by 16S rRNA gene amplicon sequencing. (a) Bray-Curtis dissimilarity of fecal microbial communities based on the relative abundances of OTUs at 97% similarity level (principal-component analysis [PCA] plots for the 12- and 24-h time points are given in Fig. S2). Dissimilarity was also calculated using ThetaYC; the result was not substantially different from that visualized by Bray-Curtis dissimilarity. (b) Relative abundances (percentage of sequences) based on the top 50 OTUs in each sample. The top 50 OTUs accounted for more than 90% of the total sequences of all AX treatment groups at all time points (relative abundances at the 12- and 24-h time points are given in Fig. S4, and relative abundances displayed in a bar graph are provided in Fig. S5). (c) Cladogram (obtained as a result of linear discriminant analysis) depicting taxa that are overrepresented in the AX samples obtained from hard and soft wheat classes compared with abundances in the initial inoculum and substrate-free blank incubations. (d) Changes in α-diversity of the fecal microbiota communities, as measured by number of species observed and inverse Simpson, Simpson, Chao, and Shannon’s index calculators (α-diversity of the fecal microbiota communities at the 12- and 24-h time points are given in Fig. S3). (e) Short-chain fatty acid (SCFA; namely, propionate, acetate, and butyrate) production by fecal microbiota at the end of the fermentation (the amounts of SCFAs produced after *in vitro* fermentation for 12 and 24 h are given in Fig. S6a; the proportions of all SCFAs produced after *in vitro* fermentation for 12, 24, and 48 h are given in Fig. S6b). Fructooligosaccharide (FOS) was used as a fast-fermenting, butyrate-producing positive control. The blank did not contain any substrate. Statistical analyses were done using two-tailed Student’s *t* test. Error bars represent the standard errors of three separate replicates.

To investigate how these differences in branch densities of AXs impact the structure and metabolism of gut microbiota, we performed an *in vitro* fermentation study using fecal microbiota obtained from three healthy donors. Microbial composition was assayed with 16S rRNA amplicon sequencing and metabolism with respect to short-chain fatty acid (SCFA) production using gas chromatography. Communities fermenting AX_SRW_ were significantly different in structure than those fermenting AX_HRS_ or AX_HRW_ based upon Bray-Curtis dissimilarity (analysis of molecular variance [AMOVA], *P < *0.001), with AX cultures from the hard varieties not distinguishable from one another (Fig. 2a and Fig. S2). These data aligned with the significant structural differences between AX_SRW_ and AX_HRS/HRW_, though not between the hard varieties. The structural variants also differentially affected community richness and evenness, with AX_SRW_ resulting in greater α-diversity indices (number of species observed, Simpson evenness, and inverse Simpson, Chao, and Shannon indices); AX_HRS_ and AX_HRW_ treatments were not distinguishable (Fig. 2d and Fig. S3). We hypothesize that the higher α-diversity values observed in AX_SRW_-fermenting cultures arose from its more complex branching structure, requiring a greater number of glycoside hydrolases for complete consumption and potentially affording more microbial niches.

10.1128/mSphere.00180-20.3 FIG S2 Bray-Curtis dissimilarity of fecal microbial communities based on the relative abundances of OTUs at 97% similarity after *in vitro* fermentation for 12 and 24 h. Download FIG S2, TIF file, 0.5 MB. Copyright © 2020 Tuncil et al. 2020 Tuncil et al. This content is distributed under the terms of the Creative Commons Attribution 4.0 International license.

10.1128/mSphere.00180-20.4 FIG S3 Changes in α-diversity of the fecal microbiota communities after *in vitro* fermentation for 12, 24, and 48 h, as measured by number of species observed and inverse Simpson, Simpson, Chao, and Shannon’s index calculators. Values with different letters in the same raw are statistically different (*P < *0.05, Tukey’s test). Fructooligosaccharide (FOS) was used as a fast-fermenting, butyrate-producing positive control. The blank did not contain any substrate. Abbreviations: HRS, arabinoxylan extracted from hard red spring wheat; HRW, arabinoxylan extracted from hard red winter wheat; SRW, arabinoxylan extracted from soft red winter wheat. Download FIG S3, TIF file, 1.8 MB. Copyright © 2020 Tuncil et al. 2020 Tuncil et al. This content is distributed under the terms of the Creative Commons Attribution 4.0 International license.

10.1128/mSphere.00180-20.5 FIG S4 Relative abundances (percentage of sequences) based on the top 50 OTUs in each sample after *in vitro* fermentation for 12 and 24 h. The top 50 OTUs accounted for more than 90% of the total sequences of all AX treatment groups at all time points. FOS was used as a fast-fermenting, butyrate-producing positive control. The blank did not contain any substrate. Download FIG S4, TIF file, 2.1 MB. Copyright © 2020 Tuncil et al. 2020 Tuncil et al. This content is distributed under the terms of the Creative Commons Attribution 4.0 International license.

10.1128/mSphere.00180-20.6 FIG S5 Relative abundances (percentage of sequences) based on the top 50 OTUs in each sample after *in vitro* fermentation for 48 h. The top 50 OTUs accounted for more than 90% of the total sequences of all AX treatment groups at all time points. FOS was used as a fast-fermenting, butyrate-producing positive control. The blank did not contain any substrate. Download FIG S5, TIF file, 2.2 MB. Copyright © 2020 Tuncil et al. 2020 Tuncil et al. This content is distributed under the terms of the Creative Commons Attribution 4.0 International license.

10.1128/mSphere.00180-20.7 FIG S6 Relative abundances (percentage of reads) of OTU20 (*Roseburia*) in each sample based on the top 50 OTUs after *in vitro* fermentation for 48 h. The top 50 OTUs accounted for more than 90% of the total sequences of all AX treatment groups at all time points. Error bars represent the standard errors of three separate replicates. SCFA, short-chain fatty acid. Download FIG S6, TIF file, 0.4 MB. Copyright © 2020 Tuncil et al. 2020 Tuncil et al. This content is distributed under the terms of the Creative Commons Attribution 4.0 International license.

AX fine structure strongly governed the relative abundances of operational taxonomic units (OTUs), such that the less branched AX_HRS_ and AX_HRW_ promoted OTU2 (*Prevotella*) and the more branched AX_SRW_ favored OTUs within genus *Bacteroides* (Fig. 2b and Fig. S4 and S5). Specifically, after 24 h of fermentation, AX_SRW_ elicited a 9.8-fold increase in the relative abundance of OTU2 but 30- and 29-fold increases in AX_HRS_ and AX_HRW_ cultures, respectively. Conversely, although fermentation of AX_HRS_ and AX_HRW_ resulted in <2.4-fold increases in the relative abundance of OTU5 (*Bacteroides*), we observed a 12.3-fold increase with AX_SRW_. AX_SRW_ also significantly increased the abundance of OTU1 (*Bacteroides*) compared with that obtained with AX_HRS_ and AX_HRW_. Linear discriminant analysis effect size (LEfSe) identified the members of family *Prevotellaceae* and subordinate taxa as discriminators for hard wheat AXs, and *Bacteroidales* and subordinate taxa were discriminators of AX_SRW_ (Fig. 1c). Both *Bacteroides* and *Prevotella* species are known to be very well equipped for the degradation of (arabino)xylans (10–14). Our findings further add to this that the structural complexity of xylans determines which of these taxa outcompete the other; *Prevotella* species seemed to better compete for less densely branched AXs but *Bacteroides* species, in general, for the more densely branched one. Moreover, in addition to *Bacteroides* and *Prevotella* species, AX consumption was shown to promote *Roseburia* species (10, 12). Our findings reveal that increases in *Roseburia* relative abundances on AX are structure dependent; neither AX_HRS_ nor AX_HRW_ significantly changed *Roseburia* relative abundances after 48 h of fermentation, whereas the fermentation of highly branched AX_SRW_ resulted in 3.2-fold increases in the abundance of *Roseburia* spp. (OTU20) (Fig. 2b and Fig. S4, S5, and S6). Collectively, these findings support the idea that soluble polysaccharides can act as “discrete fiber structures,” such that unique fiber chemical structures specifically promote particular microbial taxa in the colon (2, 8, 15).

Accordingly, structure-specific SCFA formations were observed; compared to AX_HRS_ and AX_HRW_, AX_SRW_ treatment resulted in formation of significantly higher propionate but lower acetate and butyrate concentrations (*P < *0.05) (Fig. 2e). As both the genera *Bacteroides* and *Prevotella* are regarded to increase community propiogenesis, differences in other SCFAs may stem from altered abundance or activity of acetate-consuming, butyrate-producing members of *Lachnospiraceae*. Moreover, total SCFA production occurred at a lower rate in AX_SRW_ than in hard varieties (Fig. S7a), indicating that AX_SRW_ was more slowly fermented. This was further supported by the slower decrease in pH in fermentations of AX_SRW_ than for AX_HRW/HRS_ (Fig. S8). We hypothesize that the increased branch density in AX_SRW_ may limit enzyme access to the xylan backbone until branches are partially removed, potentially allowing coordinated activity of multiple species.

10.1128/mSphere.00180-20.8 FIG S7 **(**a) Absolute amounts (mole basis) of total SCFAs, acetate, propionate, and butyrate produced after *in vitro* fermentation for 12, 24, 36, and 48 h. Total SCFA was calculated by summing up the amounts of acetate, propionate, and butyrate. (b) Proportions of acetate, propionate, and butyrate (relative to total SCFA; mole basis) produced after *in vitro* fermentation for 12, 24, 36, and 48 h. FOS was used as a fast-fermenting, butyrate-producing positive control. The blank did not contain any substrate. Error bars represent the standard errors of three separate replicates. Abbreviations: AX_HRS_, arabinoxylan extracted from hard red spring wheat; AX_HRW_, arabinoxylan extracted from hard red winter wheat; AX_SRW_, arabinoxylan extracted from soft red winter wheat. Download FIG S7, TIF file, 2.1 MB. Copyright © 2020 Tuncil et al. 2020 Tuncil et al. This content is distributed under the terms of the Creative Commons Attribution 4.0 International license.

10.1128/mSphere.00180-20.9 FIG S8 Changes in pH of the treatments after *in vitro* fermentation for 0, 6, 12, 24, 36, and 48 h. Download FIG S8, TIF file, 0.5 MB. Copyright © 2020 Tuncil et al. 2020 Tuncil et al. This content is distributed under the terms of the Creative Commons Attribution 4.0 International license.

Overall, our results demonstrate that although the general polysaccharide chemical structures obtained from closely related plant sources may seem similar, subtle structural differences significantly alter their effects on the structure and metabolic function of colonic microbiota. We submit that studies focusing on interactions among dietary fiber carbohydrates, gut microbiota, and health outcomes should include much more extensive characterization of the carbohydrate structures employed. Further, we propose that use of fibers in general categories (e.g., high- or low-fiber diets or arabinoxylan of unspecified or poorly characterized origin) without such structural data may confuse the conflicting effects of subtle structural variants, which may elicit different microbial responses.

### Data availability.

Sequence data described in this study are available in the National Center for Biotechnology Information's Sequence Read Archive under BioProject PRJNA628680 as BioSamples SAMN14749672 to SAMN14749719.
