# Supplementary material for: Subtle Variations in Dietary-Fiber Fine Structure Differentially Influence the Composition and Metabolic Function of Gut Microbiota
Source: mSphere. 2020 May 6;5(3):e00180-20. doi: 10.1128/mSphere.00180-20 (PMC7203452; doi:10.1128/mSphere.00180-20)
Supplement: TEXT S1 [file mSphere.00180-20-s0001.docx]

**Supplementary Text**

**Materials and methods**

**Arabinoxylan (AX) extractions**

AXs were extracted from wheat brans belonging to different classes of wheat; hard red winter (AXHRW), hard red spring (AXHRS), and soft red winter (AXSRW) that were generous gifts of The Mennel Milling Company (Fostoria, OH), using alkali-extraction method described (1). Briefly, wheat brans were further milled using a cyclone mill (FOSS North America, Eden Prairie, MN, USA) to obtain smaller particle sizes, which were then sieved. Wheat brans having particle size of < 500 micron were used for extractions. Wheat brans were partially defatted with hexane twice. Defatted brans (100 g) were suspended in 900 ml of water and treated with 4 ml of α-amylase (termomyl, Sigma # A3403) at 90 ± 5 °C for 20 min for the removal of starch. The samples were then subjected to protease (5 ml, Sigma #P1236) treatments at 50 ± 5 °C for 4 h for the removal of proteins, followed by drying at at 50 °C for 36 h. 50 g of enzyme treated samples were dissolved in 1 L of 1 M NaOH and heated to 60 °C under constant stirring, followed by addition of 42 ml of H2O2 and stirring for 2 h at 60 °C. The samples were centrifuged at 10000 g for 10 and supernatants were collected. AXs in the supernatants were precipitated with ethanol (1 volume of supernatant:4 volume of ethanol). AXs obtained with this procedure were shown to contain considerably high amount of starch and protein (1). Thus, AXs were subjected to another amylase and protease treatments described by Lamothe et al.(2). Briefly, AXs were dissolved in water (1:10 w/v), and the mixture were heated to 90 °C under constant stirring, followed by addition of α-amylase (termomyl, 0.2 m/g of AX). The mixture was then cooled to 60 °C and amyloglucosidase (Sigma #A7095, 0.1 ml/g of AX) was added. The mixture was mixed for 4 hours more at 60 °C. After adjusting pH to 6.0 with 1M HCl, protease (0.1 ml/g of AX) was added and left at 60 °C for 10 hours more under constant stirring. The mixture was then boiled for 20 min to inactivate the enzymes, followed by dialysis using dialysis bag 6-8 kDa cut-off for at least 36 h and freeze-dried.

After the second amylase and protease treatment, AXs samples were subjected to upper gastrointestinal digestion protocol, [extensively described by Tuncil et al.(3) - method A] in order to make sure that all of the digestible components were removed before inoculation with fecal microbiota for *in vitro* fermentation experiment. These samples were used for further analyses.

**Molecular weight and size**

The samples were dissolved in purified water (2 mg/ml). Molecular weight and size distribution of AXs were determined using a high-performance size exclusion chromatograph equipped with multi-laser scattering and refractive index detectors (HPSEC-MALS-RI) [a DAWN DSP-F laser photometer fitted with argon laser at λ = 488 nm with a K-5-129 flow cell (Wyatt Technology, Santa Barbara, CA) and an Optilab 903 interferometric refractometer (Wyatt Technology, Santa Barbara, CA)], a pump (model LC-10AT vp, Shimadzu Corp., Columbia, MD), and syringe sample loading injector (model 7125, Rheodyne Inc., Catati, CA) as previously reported (4,5). Molecular size calculations and data processing was done using ASTRA software (Version 4.9, Wyatt Technology, Santa Barbara, CA).

**Total Starch Content**

Total starch contents of the samples were quantified spectrophotometrically using total starch assay kit (Magazyme, Wicklow, Ireland # K-TSTA) according to manufacturer's instruction.

**Monosaccharide composition**

AX samples were first subjected to carboxyl reduction, as previously described (6), in order to pre-reduce the uronic acids to their native neutral sugar counterparts. Pre-reduced samples were hydrolyzed with trifluoroacetic acid (Sigma #T6508), followed by reduction with sodium borodeuteride (Sigma #205591) and acetylation with acetic anhydride (Sigma #320102), as previously described (7). The monosaccharides were then quantified as their alditol acetate derivatives on a capillary column (SP2330; SUPELCO, Bellefonte, PA) by gas chromatography coupled with mass spectrometry (GC/MS; models 7890A and 5975C inert MSD with a Triple-Axis detector, Agilent Technologies, Inc., Santa Clara, CA). The GC/MS set up conditions were given elsewhere (3,8). The proportions of uronic acid/native sugar was determined as the mean ratios of corresponding m/z fragments, as previously described (9).

**Glycosyl-linkage profiles**

AX samples were first subjected to carboxyl reduction using carbodiimide reagent (Sigma #C106402) and sodium borodeuteride (Sigma #205591), as previously described (6), in order to pre-reduce the uronic acids to their native neutral sugar counterparts. Pre-reduced samples were partially methylated with iodomethane (Sigma #289566), followed by hydrolyzation with trifluoroacetic acid, and reduction with sodium borodeuteride and acetylation with acetic anhydride, as previously described (7). Partially methylated alditol acetates were run on GC/MS coupled with SP-2330 column (3,8) and quantified as previously specified (10).

***In vitro* fermentation**

*In vitro* fecal fermentation was performed in an anaerobic chamber (Coy Laboratory Products, Inc., Grass Lake,MI, U.S.A.) supplied with a 90% N2, 5% CO2, and 5% H2 gas mixture. 44 ± 1 mg of AXs samples were weighed in Balch Tubes Chemglass Life Sciences, Vineland, NJ) for each time points (12, 24, 26, and 48 h). We transferred the tubes containing AXs, fructooligosaccharide (FOS; Sigma-Aldrich, St. Louis, MO) (positive control), and blank (negative control) in the anaerobic chambers and equilibrated them with the chamber atmosphere overnight. For all experiments, we used the previously-described phosphate-buffered gut mineral medium containing trace elements(3). The media was placed in the anaerobic chamber overnight to remove oxygen and resazurin was used as the oxygen indicator.

The next day, 4 ml of gut mineral medium was added to each tube containing AXs and FOS samples, and the blank. We collected and pooled fecal samples from 3 healthy donors (2 males, 27 and 32 years old, respectively; 1 female, 31 years old, all three were omnivores) who were consuming their routine diets and had not taken antibiotics for at least 3 months. Fecal samples were tightly sealed in plastic tubes, kept on ice prior to rapidly being transferred into the anaerobic chamber, and used within 2 h of collection. The fecal samples were homogenized gut mineral media in the ratio 1:10 (w/v), followed by filtration through four layers of cheese cloth. Filtered fecal slurries were pooled in equal ratios, and then 0.4 ml of pooled fecal slurry was inoculated into each tube. Pooling fecal slurries is a common method to investigate how variations in the fine structures of dietary components impact the colonic microbiota (1,11-14), providing a diverse initial pool of species that is not constrained by the idiosyncratic gut microbiomes of individuals. Use of pools of fecal microbiota for *in vitro* studies results in similar microbial community profiles and activities compared to those obtained from single donors (15). The tubes were then immediately closed with butyl rubber stoppers (Chemglass Life Sciences), sealed with aluminum seals (Chemglass Life Sciences), and incubated at 37 oC in a shaking incubator (Innova 42, New Brunswick Scientific, Edison, NJ) at 150 rpm at an approximately 45° angle. All analyses were performed in triplicate. Human stool collection and use protocols were reviewed and approved by Purdue University’s Institutional Review Board (IRB Protocol #1701018645).

**Sample collection for SCFA and DNA analysis**

At each time point, two aliquots were collected from each tube for DNA extraction (1 ml) and SCFA analysis (0.4 ml). Samples collected for DNA extraction were immediately stored at -80 °C until further analysis. 100 ml of an internal standard mixture (prepared by combining 157.5 µl of 4-methylvaleric acid, 1.47 ml of 85% phosphoric acid, 39 mg of copper sulfate pentahydrate in a final volume of 25 ml ultrapure water) were immediately added to samples collected for SCFA analysis, which were then vortexed and stored at -80 oC until analysis.

**SCFA analysis**

SCFA analyses were performed as previously described (3,8). Briefly, the samples were thawed at room temperature and centrifuged at 13,000 rpm for 10 mins. Supernatants (4 µl) were analyzed using a gas chromatography (GC-FID 7890A; Agilent Technologies Inc.) on a fused silica capillary column (NukonTM SUPELCO No: 40369-03A, Bellefonte, PA) under the following conditions: Injector temperature at 230 oC; initial oven temperature at 100 oC; temperature increase of 8 oC/min to 200 oC with a hold for 3 min at final temperature. Helium was used as a carrier gas at 0.75 ml/min. Acetate (catalog number: A38S), propionate (catalog number: A258), and butyrate (catalog number: AC108111000) purchased from Fisher Scientific (Hampton, NH) were used as external standards. 4-methylvaleric acid (catalog number: AAA1540506, Fisher Scientific) was used as an internal standard for quantification.

**DNA extraction**

The DNA extraction from the samples were carried out using FastDNA SPIN® kit for Feces (product code: 116570200) according to the manufacturer’s instructions (MP Biomedical, Santa Ana, USA) with the minor modification described by Tuncil et al. (2018a; 2018b).

**16S rRNA sequencing**

16S rRNA sequencing was performed as previously described (3,8,16). Briefly, the V4-V5 region of the 16S rRNA gene was amplified by PCR using the universal bacterial primers: 515-FB (GTGYCAGCMGCCGCGGTAA) and 926-R (CCGYCAATTYMTTTRAGTTT) 16. The PCR solution included 2 µl of template DNA (containing > 5 ng/µl DNA), 0.625 µmole of each primer, 12.5 µl of HiFi hot start ready mix (Kapa Biosystems, Wilmington, MA # KK2602), and 0.016 µg of BSA (Fisher Scientific # BP9706100,), in a buffer containing Tris-HCl (final concentration in the reaction 0.8 mM; Fisher Scientific #BP1758,), KCl (final concentration 4 mM; Fisher Scientific #P217,), EDTA (4 µM final concentration; Fisher Scientific #S311) and glycerol (final concentration 1.6 %, Fisher Scientific #G33,) in a final reaction volume of 25 µL. The cycling parameters were as the following: An initial denaturation at 98 °C for 3 minutes, followed by 22 cycles of denaturation at 98 °C for 10 seconds, annealing at 50 °C for 30 seconds and extension at 72°C for 30 seconds. Unincorporated dNTPs and primers were removed using the Axygen AxyPrep PCR Clean-up Kit (Axygen Scientific, an imprint of Corning Life Sciences, Tewksbury, MA) according to the manufacturer’s instructions, followed by barcoding the PCR products using the TruSeq dual-index approach and purified again using the AxyPrep PCR Clean-up Kit. Barcoded samples were quantitated via Qubit dsDNA HS Assay Kit (Invitrogen, Carlsbad, CA), and pooled. Quality control for pools was performed by running 1 µL of each pool on an Agilent Bioanalyzer with a High Sensitivity Chip (Agilent, Santa Clara, CA) and then quantified the the pool loading via the KAPA Library Quantitation Kit for Ilumina platforms. Sequencing was performed using on an Illumina MiSeq run with 2 x 250 cycles and V2 chemistry (Illumina, Inc., San Diego, CA) at the Purdue Genomics Core Facility.

**Sequence processing and community analysis**

Sequences were processed using mothur v.1.39.3 according to the MiSeq SOP (<https://www.mothur.org/wiki/MiSeq_SOP>) (17,18) with the modifications as previously described (3,8). OTU classifications at the species level are reported as the percentage of reads that were classified within a given species at a bootstrap value of 95 % or greater. α-Diversity indices were calculated using the nseqs, coverage, invsimpson, simpsoneven, chao, and shannon calculators and β-diversity metrics were calculated using the braycurtis and thetayc calculators as implemented in mothur. Distance matrices based upon β-diversity metrics were plotted for visualization using the pcoa command in mothur (18). Analysis of molecular variance (AMOVA) tests were also computed between samples using the amova command in mother (18-21) in order to determine whether centroids were significantly different. LEfSe-formatted files were generated using mothur using make.lefse, and linear discriminant analysis was performed using LEfSe v.1.6 (22).

**Statistical analyses**

Data are presented as mean±SEM. Statistical analyses were performed using GraphPad Prism version 8.0.1 for Mac OS X (GraphPad Software, Inc. La Jolla, CA). Analysis of variance (ANOVA) was performed at α = 0.05 significance level to determine differences among the samples and controls (for **Figs. S3** and **S6b**). Tukey’s multiple comparison test at α = 0.05 was used to see whether mean differences were statistically different (for **Figs. S3** and **S6b**). For other data (Figs. 1, 2, S1, and Table S1), statistical analyses were performed using two-tailed, unpaired students' t-test at α = 0.05 significance level.

**References**

1 Rose DJ, Patterson JA, Hamaker BR. 2010. Structural differences among alkali-soluble arabinoxylans from maize (Zea mays), rice (Oryza sativa), and wheat (Triticum aestivum) brans influence human fecal fermentation profiles. Journal of Agricultural and Food Chemistry 58: 493-499, doi:10.1021/jf9020416.

2 Lamothe LM, Srichuwong S, Reuhs BL, Hamaker BR. 2015. Quinoa (Chenopodium quinoa W.) and amaranth (Amaranthus caudatus L.) provide dietary fibres high in pectic substances and xyloglucans. Food Chemistry 167: 490-496, doi:10.1016/j.foodchem.2014.07.022.

3 Tuncil YE, Thakkar RD, Arioglu-Tuncil S, Hamaker BR, Lindemann SR. 2018. Fecal microbiota responses to bran Particles are Specific to cereal type and *in vitro* digestion methods that mimic upper gastrointestinal tract passage. Journal of Agricultural and Food Chemistry 66: 12580-12593, doi:10.1021/acs.jafc.8b03469.

4 Tuncil YE et al. 2017. Reciprocal prioritization to dietary glycans by gut bacteria in a competitive environment promotes stable coexistence. mBio 8: e1068-1017.

5 Fang F. et al. 2019. Shear-thickening behavior of gelatinized waxy starch dispersions promoted by the starch molecular characteristics. International Journal of Biological Macromolecules 121: 120-126, doi:10.1016/j.ijbiomac.2018.09.137.

6 Carpita NC, McCann MC. 1997. Some new methods to study plant polyuronic acids and their esters. In: Townsend R, Hotchkiss A (eds). Progress in Glycobiology. Marcell Dekker: New York, NY. pp 595-611.

7 Pettolino FA, Walsh C, Fincher GB, Bacic A. 2012. Determining the polysaccharide composition of plant cell walls. Nature Protocols 7, 1590-1607, doi:10.1038/nprot.2012.081.

8 Tuncil YE, Thakkar RD, Marcia ADR, Hamaker BR, Lindemann SR. 2018. Divergent short-chain fatty acid production and succession of colonic microbiota arise in fermentation of variously-sized wheat bran fractions. Scientific Reports, 10.1038/s41598-41018-34912-41598, doi:10.1038/s41598-018-34912-8.

9 Kim JB, Carpita NC. 1992. Changes in esterification of the uronic acid groups of cell-wall polysaccahrides during elongation of maize coleoptiles. Plant Physiology 98: 646-653, doi:10.1104/pp.98.2.646.

10 Naran R, Chen GB, Carpita NC. 2008. Novel rhamnogalacturonan I and arabinoxylan polysaccharides of flax seed mucilage. Plant Physiology 148: 132-141, doi:10.1104/pp.108.123513.

11 Rumpagaporn P, et al. 2015. Structural features of soluble cereal arabinoxylan fibers associated with a slow rate of *in vitro* fermentation by human fecal microbiota. Carbohydrate Polymers 130: 191-197, doi:10.1016/j.carbpol.2015.04.041.

12 Kaur A, Rose DJ, Rumpagaporn P, Patterson JA, Hamaker BR. 2011. *In vitro* batch fecal fermentation comparison of gas and short-chain fatty acid production using "slowly fermentable" dietary fibers. Journal of Food Science 76: H137-H142, doi:10.1111/j.1750-3841.2011.02172.x.

13 Aguirre M, Jonkers D, Troost FJ, Roeselers G, Venema K. 2014. *In vitro* characterization of the impact of different substrates on metabolite production, energy extraction and composition of gut microbiota from lean and obese subjects. Plos One 9: doi:10.1371/journal.pone.0113864.

14 Kortman GAM et al. 2016. Microbial metabolism shifts towards an adverse profile with supplementary iron in the TIM-2 *in vitro* model of the human colon. Frontiers in Microbiology 6: doi:10.3389/fmicb.2015.01481.

15 Aguirre M, Ramiro-Garcia J, Koenen ME, Venema, K. 2014. To pool or not to pool? Impact of the use of individual and pooled fecal samples for *in vitro* fermentation studies. Journal of Microbiological Methods 107: 1-7, doi:10.1016/j.mimet.2014.08.022.

16 Walters W, et al. 2016. Improved bacterial 16S rRNA gene (V4 and V4-5) and fungal internal transcribed spacer marker gene primers for microbial community surveys. Msystems 1: doi:10.1128/mSystems.00009-15.

17 Kozich JJ, Westcott SL, Baxter NT, Highlander SK, Schloss PD. 2013. Development of a dual-index sequencing strategy and curation pipeline for analyzing amplicon sequence data on the MiSeq Illumina Sequencing Platform. Applied and Environmental Microbiology 79: 5112-5120, doi:10.1128/aem.01043-13.

18 Schloss PD et al. 2009. Introducing mothur: Open-source, platform-independent, community-supported software for describing and comparing microbial communities. Applied and Environmental Microbiology 75: 7537-7541, doi:10.1128/aem.01541-09.

19 Excoffier L, Smouse PE, Quattro JM. 1992. Analysis of molecular variance inferred from metric distances among DNA haplotypes - Application to human motochondrial-DNA restriction data. Genetics 131: 479-491.

20 Anderson MJ. 2001. A new method for non-parametric multivariate analysis of variance. Austral Ecology 26: 32-46, doi:10.1046/j.1442-9993.2001.01070.x.

21 Martin AP. 2002. Phylogenetic approaches for describing and comparing the diversity of microbial communities. Applied and Environmental Microbiology 68: 3673-3682, doi:10.1128/aem.68.8.3673-3682.2002.

22 Segata N, et al. 2011. Metagenomic biomarker discovery and explanation. Genome Biology 12: doi:10.1186/gb-2011-12-6-r60.
