# Supplementary figures and images for: Subtle Variations in Dietary-Fiber Fine Structure Differentially Influence the Composition and Metabolic Function of Gut Microbiota
Source: mSphere. 2020 May 6;5(3):e00180-20. doi: 10.1128/mSphere.00180-20 (PMC7203452; doi:10.1128/mSphere.00180-20)

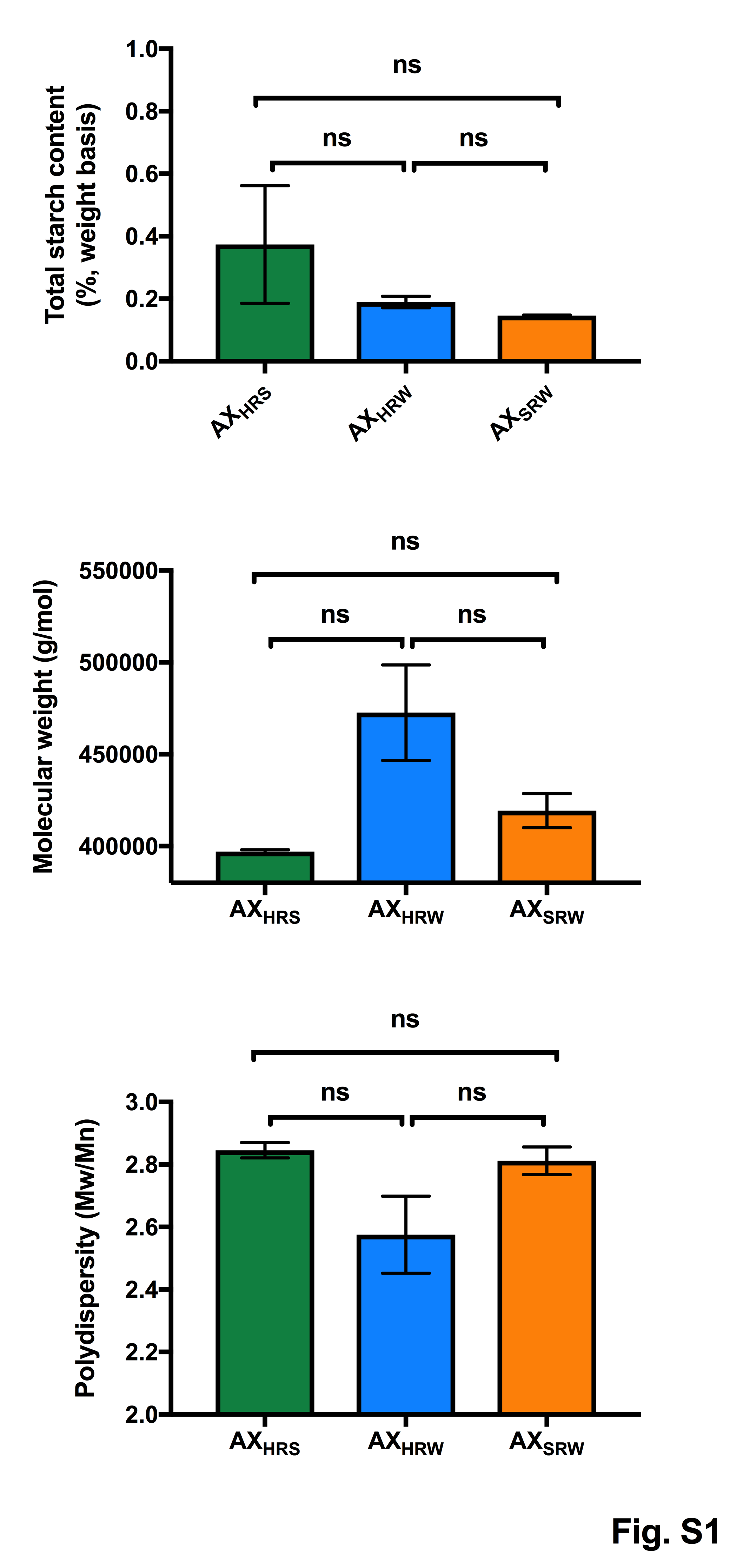

Supplement: FIG S1 [file mSphere.00180-20-sf001.tif]

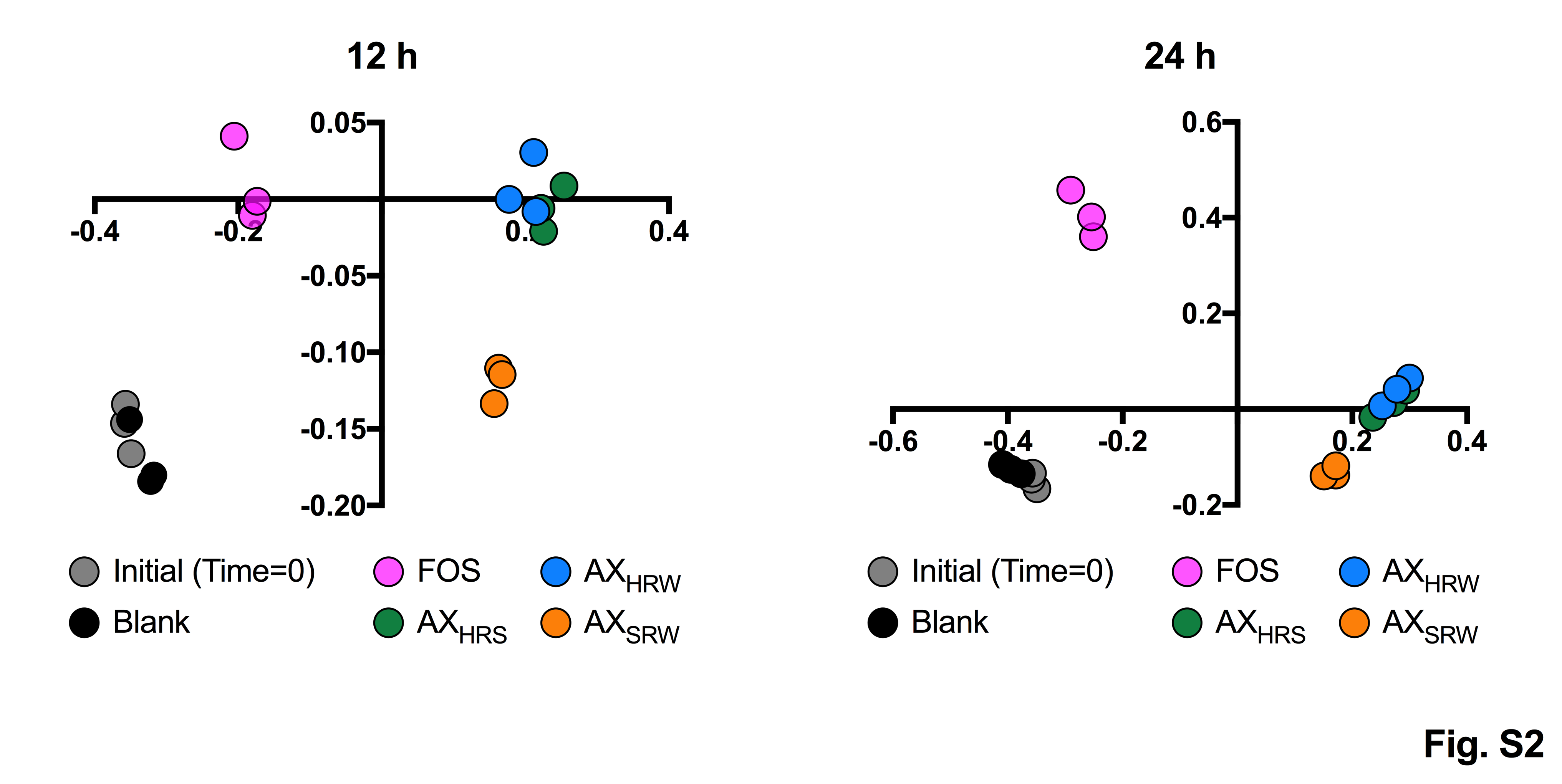

Supplement: FIG S2 [file mSphere.00180-20-sf002.tif]

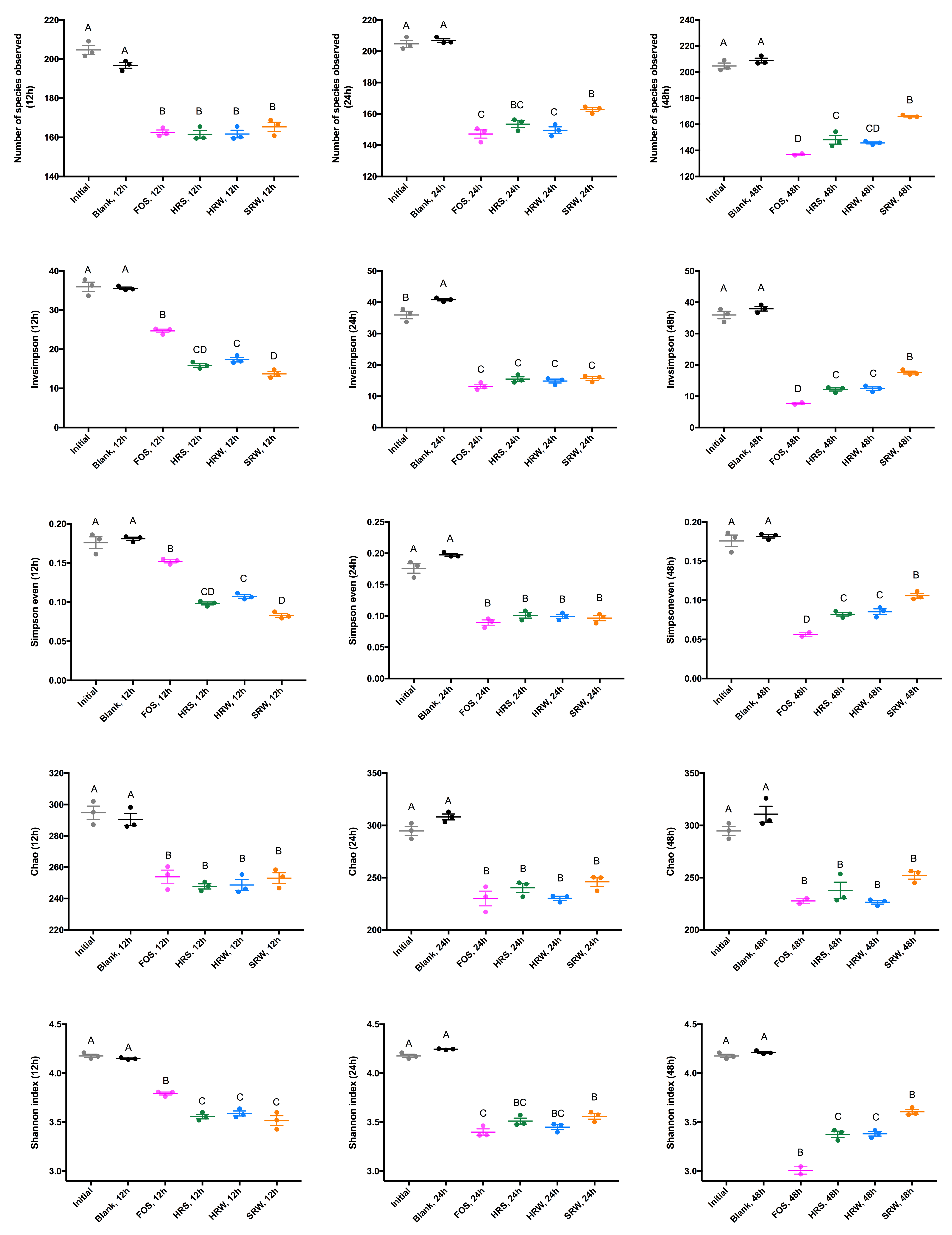

Supplement: FIG S3 [file mSphere.00180-20-sf003.tif]

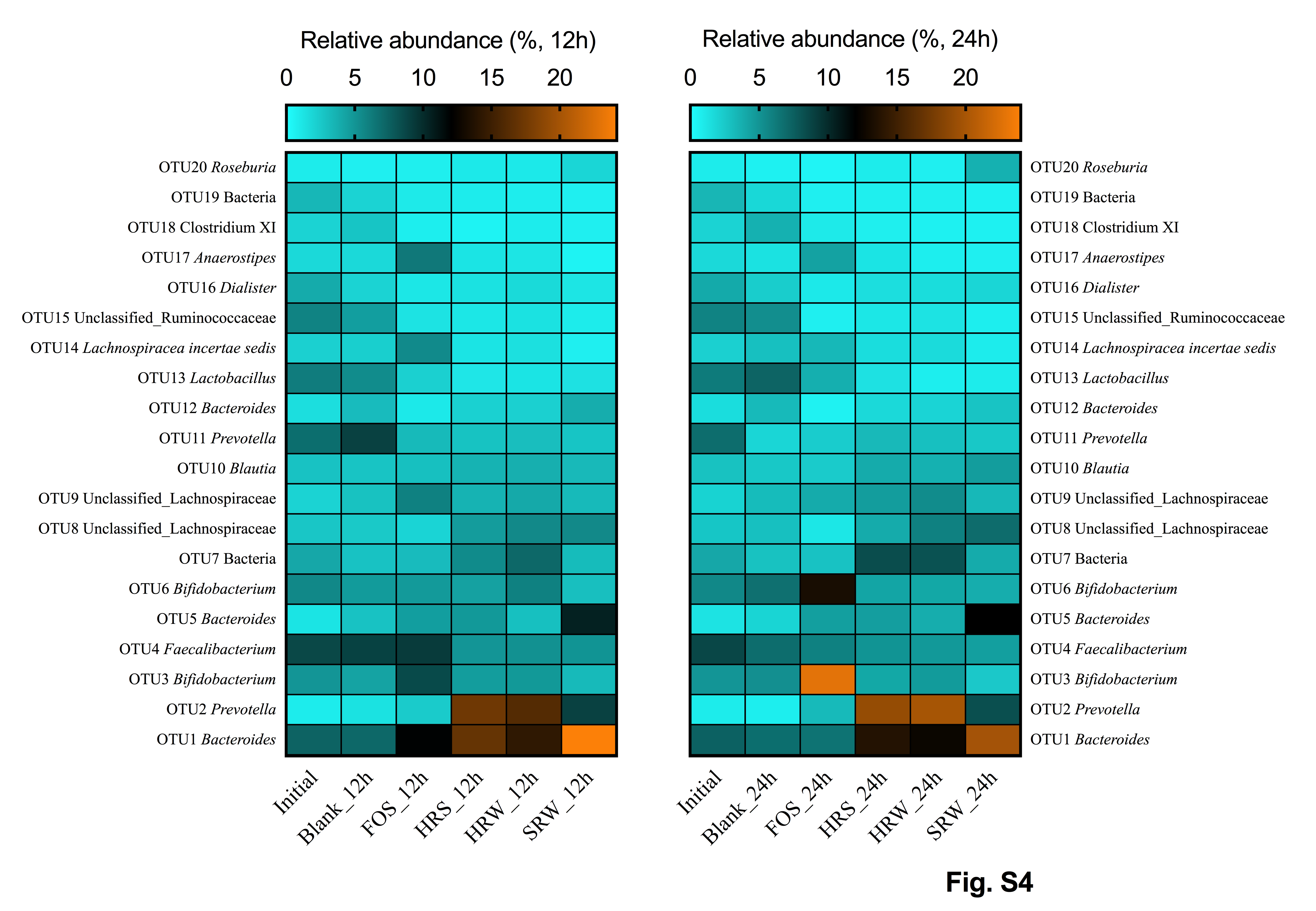

Supplement: FIG S4 [file mSphere.00180-20-sf004.tif]

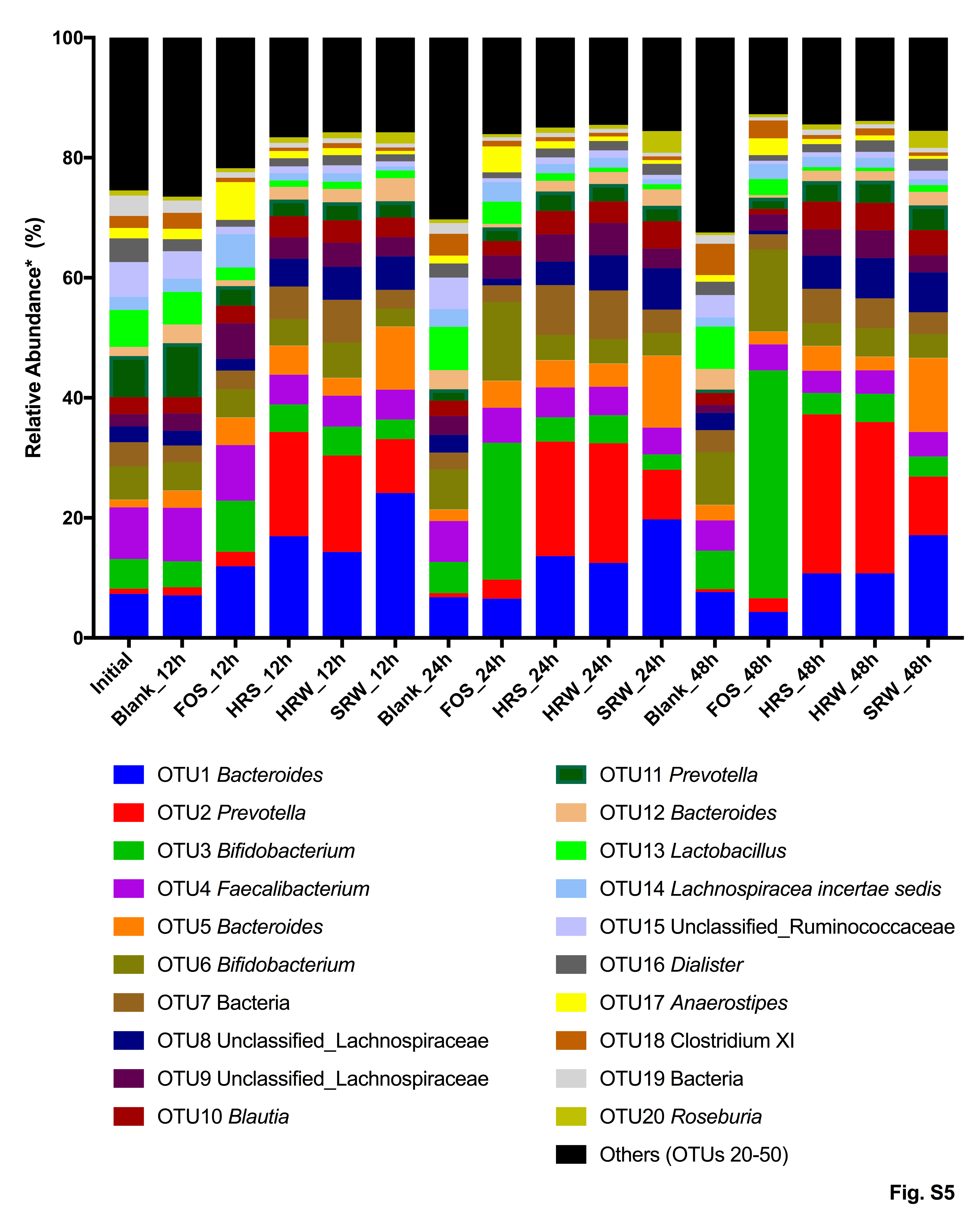

Supplement: FIG S5 [file mSphere.00180-20-sf005.tif]

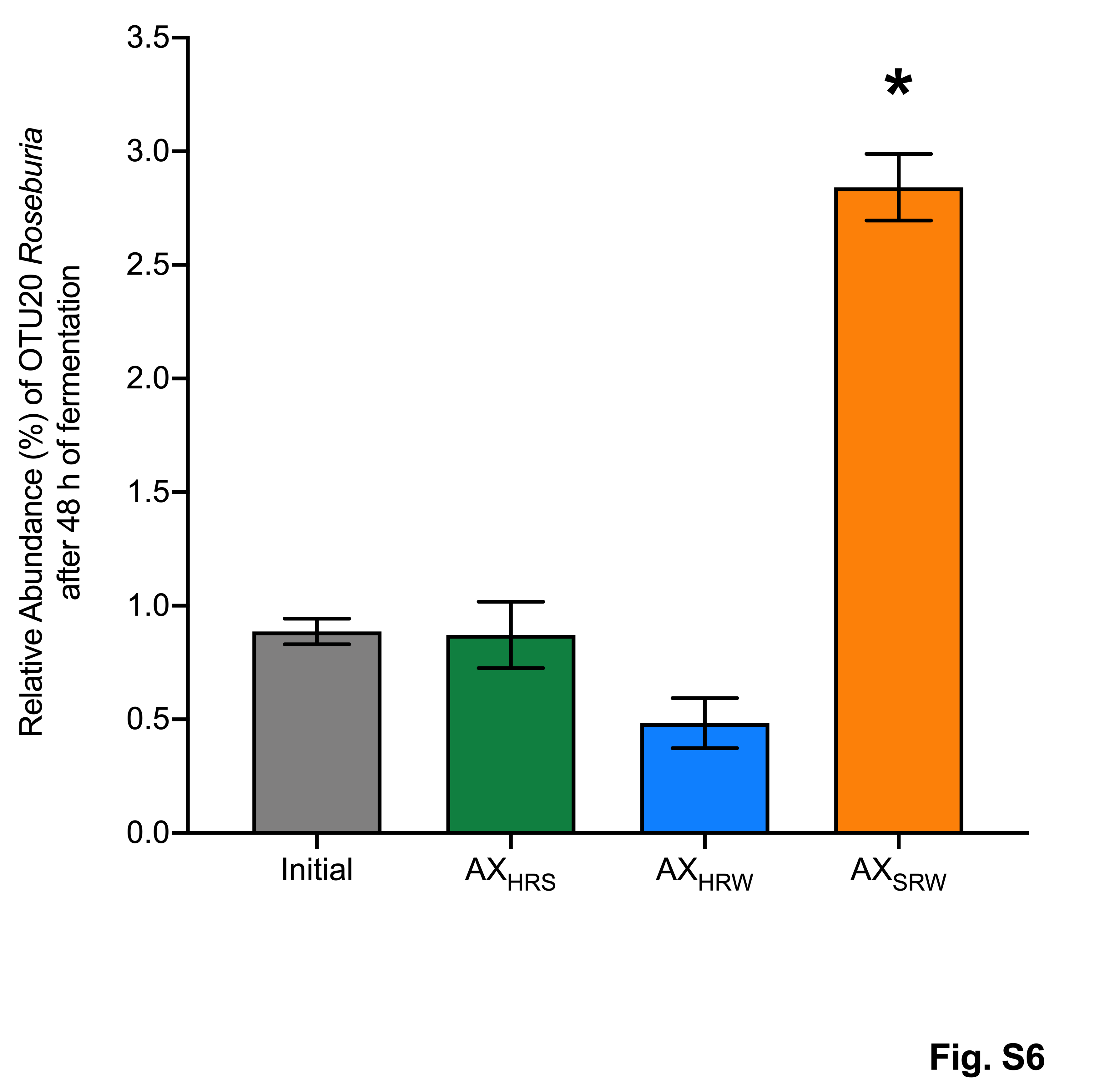

Supplement: FIG S6 [file mSphere.00180-20-sf006.tif]

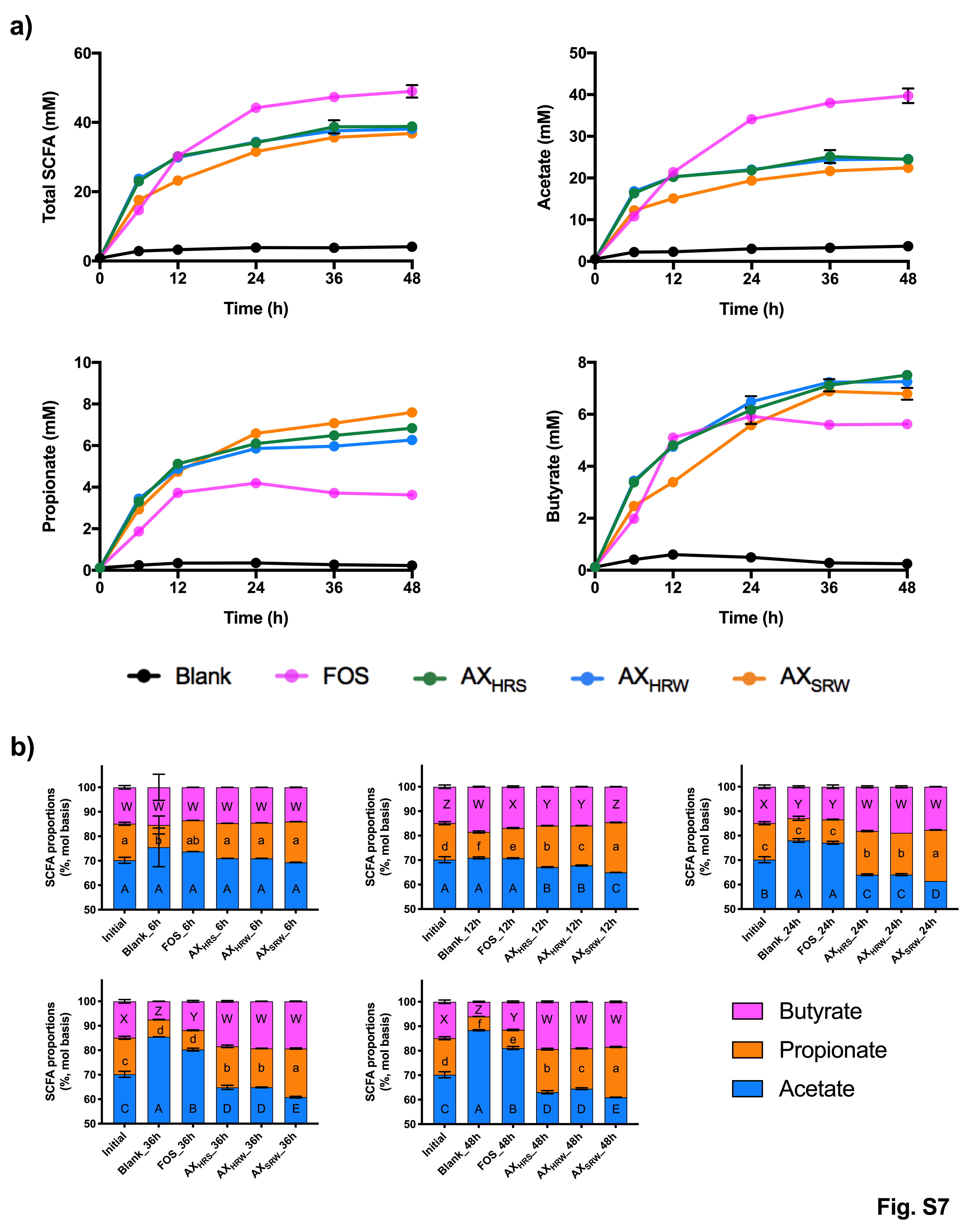

Supplement: FIG S7 [file mSphere.00180-20-sf007.tif]

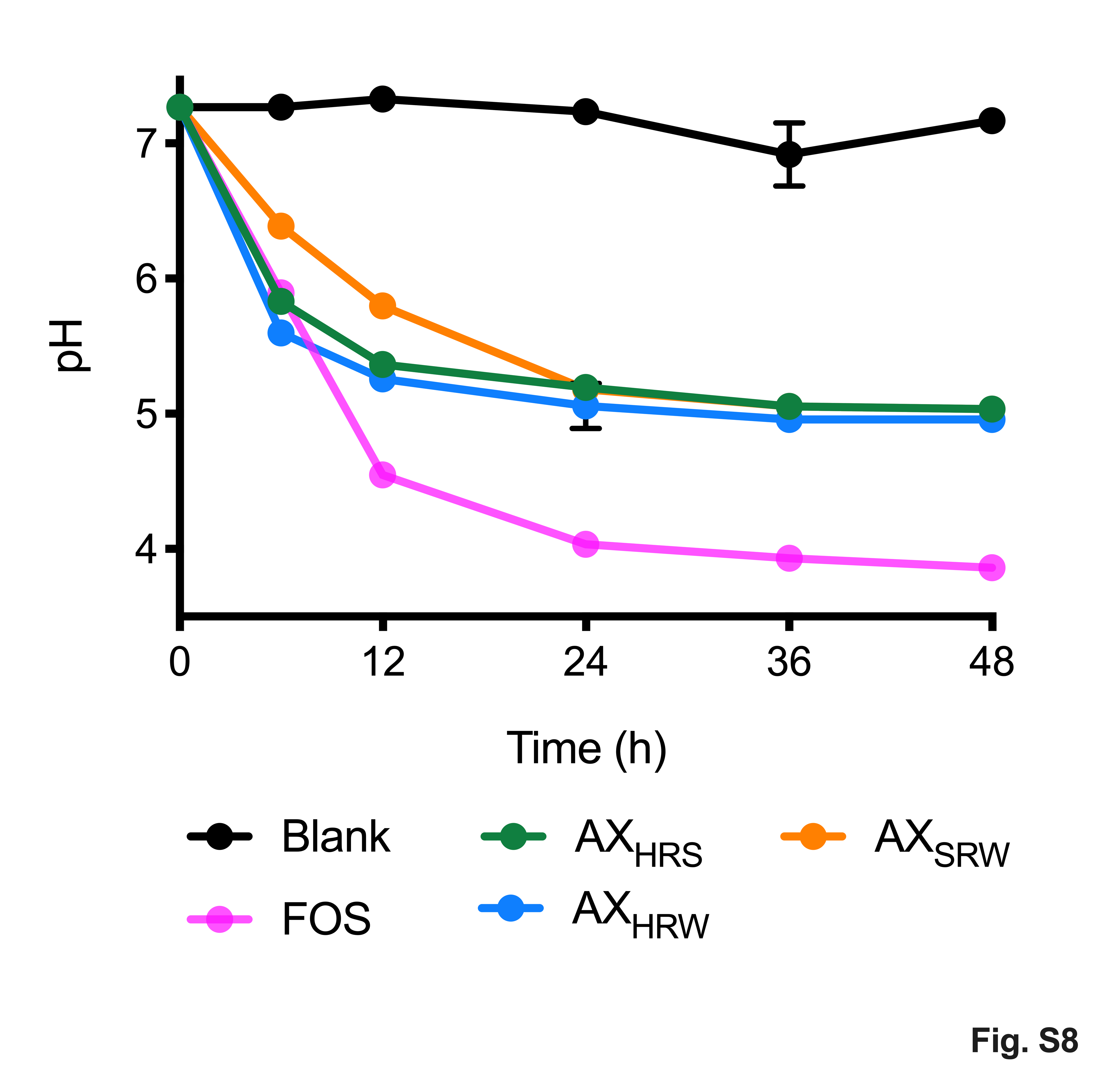

Supplement: FIG S8 [file mSphere.00180-20-sf008.tif]
