# Supplementary material for: Subtle Variations in Dietary-Fiber Fine Structure Differentially Influence the Composition and Metabolic Function of Gut Microbiota
Source: mSphere. 2020 May 6;5(3):e00180-20. doi: 10.1128/mSphere.00180-20 (PMC7203452; doi:10.1128/mSphere.00180-20)
Supplement: TABLE S1 [file mSphere.00180-20-st001.docx]

| Linkage Type/Sample | AX_HRS_ | AX_HRW_ | AX_SRW_ |
| --- | --- | --- | --- |
| t-Ara(f) | 27.83 | 29.48 | 27.44 |
| t-Ara(p) | 4.39 | 4.26 | 6.07 |
| 2-Ara(f) | 1.77**^ab^** | 1.32**^b^** | 2.45**^a^** |
| 3-Ara(f) | 4.10**^b^** | 4.06**^ab^** | 6.45**^a^** |
| 4-Ara(p) | 1.02**^a^** | 0.57**^b^** | 0.85**^ab^** |
| 4-Xyl | 42.79**^a^** | 39.83**^a^** | 32.86**^b^** |
| 3-4-Xyl | 7.93 | 9.80 | 10.31 |
| 2-3-4-Xyl | 4.44**^b^** | 5.20**^ab^** | 6.09**^a^** |
| t-Rha | 0.21**^b^** | 0.19**^b^** | 0.39**^a^** |
| t-Glu | 0.17 | 0.08 | 0.30 |
| t-GluA | 1.07 | 0.98 | 1.61 |
| t-Gal | 1.68 | 1.19 | 1.97 |
| t-GalA | 0.70 | 0.83 | 1.05 |
| 4-Gal | 0.87 | 1.22 | 1.05 |
| 4-GalA | nd | nd | 0.28 |

**Table S1.** Linkage compositions of AXs (%, mol basis)*

* **Abbreviations**: **t:** terminal; **f:** furanosyl ; **p:** pyronyl;

**Ara:** Arabinose; **Xyl:** Xylose; **Rha:** Rhamnose; **Glu:** Glucose;

**GluA:** Glucuronic acid; **Gal:** Galactose; **GalA:** Galacturonic

acid; nd: not detected. Values devoted with different letters in

the same raw are statistically different (P < 0.05, two-tailed

student’s t-test). No significant difference was observed

between values in a raw that are not devoted with a letter.
